# Enabling Green Crowdsourced Social Delivery Networks in Urban Communities †

**DOI:** 10.3390/s22041541

**Published:** 2022-02-17

**Authors:** Kevin Choi, Luca Bedogni, Marco Levorato

**Affiliations:** 1 Donald Bren School of Information and Computer Science, University of California, Irvine, CA 92697, USA; kwchoi1@uci.edu (K.C.); levorato@uci.edu (M.L.); 2 Department of Physics, Informatics and Mathematics, University of Modena and Reggio Emilia, 41125 Modena, Italy

**Keywords:** mobile crowdsensing, smart city, performance evaluation

## Abstract

With the ever-increasing popularity of wearable devices, data on the time and location of popular walking, running, and bicycling routes is expansive and growing rapidly. These data are currently used primarily for route discovery and mobile context awareness, as it provides precise and updated information about urban dynamics. We leverage these data to build ad hoc transportation flows, and we present a novel model that creates delivery networks from these zero-emission transportation flows. We evaluate the model using data from two popular datasets, and our results indicate that such networks are indeed possible, and can help reduce traffic, emissions, and delivery times. Moreover, we demonstrate how our results can be consistently reproduced in different cities with different subsets of carriers. We then extend our work into predicting routes of vehicles, hence possible delivery flows, based on the traces history. We conclude this paper by laying the groundwork for a future real-world study.

## 1. Introduction

In the last decade, the last mile challenge has been the study of many different works. Academic and industrial research analyzed how to perform it in an efficient and sustainable way [1] colorred, to optimize transportation and eventually the delivery itself. Certainly, there have been many different improvements, which have been proposed and implemented in order to make non-sustainable longer deliveries greener, more efficient and more cost effective. However the last leg of the delivery, which is often called “the last mile”, has received less improvements over time, as it still uses the usual centric traditional delivery model, in which a logistic operator collects all the parcels from the logistic center and delivers them to the final customer. It has been shown that such model is unpractical and non-sustainable, as it needs larger carriers to collect all the parcels, which may be unfeasible specifically in constrained areas [1,2]. There are different studies which estimate the last mile to be roughly 28% of the total cost of the entire delivery [3,4], while also raising problems such as pollution, cost, traffic and many others [5]. There have been a number of different proposals in the literature and in different projects which tackled this problem, such as delivery lockers, as they offer an intermediate point accessible to the end customer. Unfortunately, their adoption requires to build the lockers, which is challenging particularly in dense cities.

There have also been proposals, closer to the work on which we focused, which leverage existing routes already traveled by citizens, to make them deliver packages covering the last mile. At first, this proposal does not increase the traffic, as those routes would be traveled by users anyway hence they can be re-utilized also for other purposes. This could be a cost effective solution particularly for smaller packages, as they could be also carried by people bicycling or simply walking. These parcels may include food, small retail items, books and so on. These kind of delivery methods need to be crowdsourced, to open up social platforms which can recruit users to perform specific deliveries, and would also open up interesting scenarios which are currently difficult to perform for logistic operators, such as indoor deliveries, car limited areas, and in inaccessible areas. For logistic operators, adding those services to their offer would increase the cost, which would eventually be reflected on the delivery cost asked to the users. Crowdsourcing and leveraging existing routes avoid these aforementioned problems, while still providing the service in a cost-effective way and without increasing pollution or traffic.

College campuses could also be another interesting scenario for this kind of deliveries, as they encompass all the peculiar characteristics needed for such scenario to work. There are a number of potential users, mostly traveling with bikes or by foot, and the routes are highly heterogeneous, both in space and in time. Moreover, scenarios such as college campuses also provide different challenges as we already mentioned, such as car free zones, indoor environments and routes which can be predicted for instance accounting for lecture hour, which are some of the key characteristics to realize ad hoc delivery networks [6,7]. In fact, campuses provide a more constrained areas compared to larger cities, hence there may be more connections thus more opportunities for delivery.

It is evident that this specific scenario carries several challenges: since users travel in and out of buildings and around larger areas such as cities, it is difficult to track their movements to efficiently deploy the ad hoc delivery network. In larger and highly populated areas for instance users may have more constrained routes, without the freedom to deviate from their original intended journey, which may limit such networks.

In our study, we provide the following novelties over the state of the art: *(i)* we present a novel model for crowdsourced ad hoc delivery networks aimed at increasing the efficiency of the network without introducing additional traffic and pollution; *(ii)* we study our system on real data for two different datasets, one in New York City and the other one on the UCI Campus; *(iii)* we assess similarities between different environments and architectures leveraging an objective metrics-based approach, to be able to generalize our results.

As a note, for the remainder of the paper who submits a delivery request will be named *user*. Depending on the scenario, the party taking over the delivery will be named *driver*. When instead dealing with a non motorized scenario, the same entity will be instead named *runner*, and in the case we need to mention them both as the same time we will use the term *carrier*.

The rest of this paper is structured as follows: in Section 2 we present and discuss related studies from the literature; Section 3 describes instead the model we have developed and used within this study; Section 4 presents our quantitative and qualitative results that we obtained running our system on two real datasets; Section 5 presents results related to the predictability of future routes by carriers; Section 6 builds the framework for a real-world delivery system and Section 7 concludes our study, discussing our findings and presenting future works on this topic.

## 2. Related Work

Work on solving the last mile challenge can be traced as far back as 1999 with one of the earliest works proposing a delivery box [8]. A decade later, ref. [2] improved on the delivery box with increased modularity and security through one-time passwords. The paper culminated in the CityLog project that placed several of these concept delivery boxes in Berlin, Lyon, and Turin. Around the same time, Amazon began piloting its Amazon locker program in six metropolitan cities including Seattle, San Francisco, and New York. Ref. [9] studies how to use drones to perform last-mile deliveries. The rationale is that drones may serve better hard to reach areas, although being limited in range and potentially carrying less weight compared to other popular delivery methods. In [10], it is studied more broadly the user acceptance on autonomous delivery vehicles, specifically in Germany. Apart from autonomus vehicles deliveries, it has also been proposed the methodologies presented in [11], where shared vehicles are used to perform last mile delivery. The overarching idea is to combine deliveries and pickups, so that delivery paths are optimized.

As we already mentioned, academia and industry studied many different solutions, also cooperating between each other, to solve the last mile challenge. Among these solutions, it is worth to mention [12], which proposed a freight-tricycle system which was designed and deployed specifically for Manhattan. Such system leveraged the urban dynamics of Manhattan, as well as its widely available bike sharing system. After running the system for some time, which was also needed to let users adapt to the new service, there have been different statements labeling the study as a success, such as a New York Times article (https://www.nytimes.com/2019/12/04/nyregion/nyc-cargo-bikes-delivery.html, accessed on 1 December 2021). It is also worth to note the more recent study which was presented in [13], where the authors of the work identified potentials and limits of crowdsourced delivery networks, while also discussing possible improvements and research challenges to effectively realize such vision. Similarly to that, ref. [14] focused on how to leverage taxis as delivery vehicles, which has been the focus of many recent industry startups like UberRUSH [15], Postmates, and Deliv. Among these, Postmates tried to achieve zero-emissions by launching zero-emissions crowdsourced delivery networks in the areas of San Francisco, New York, Portland, and Seattle. The zero emission network leverages on the use of electric bicycles. Ref. [16] further cements the idea of utilizing bike-share systems for crowdsourced deliveries by analyzing user satisfaction when riders are incentivized for deviating from their routes to make deliveries.

Closer to our work, we cite [17], which analyzes and show the benefits of crowdsourced zero-emission delivery networks. In such work the authors mention the different advantages that such network may provide, including pollution, noise, and in certain cases efficiency over traditional delivery network.

In [18], the authors leverage social relations among different users to share delivery operations, and they also present a survey study aimed at analyzing people’s attitude towards ad hoc social delivery networks, which is fundamental to understand whether such networks may be actually deployed. Finally [4] models the last mile delivery challenge as a standalone, crowdsourcing problem, where the primary objective is to minimize computational cost.

Most recently, interest in sustainable crowdsourced delivery has increased dramatically. Two of the most promising works to come out in 2021 are [19,20]. The first work excellently summarizes both the incredible potential of sustainable crowdsourced delivery and its challenges while the second work proposes a simulation framework that can be used by all current and future proposed delivery models, including our own, to objectively evaluate their effectiveness.

## 3. Feasibility Study

To analyze the performance of our proposed crowdsourced delivery network, we perform two different studies, a part of which has been extended from [21]. The first one leverages on the wide availability of New York taxi data, so we are able to simulate an ideal environment for the delivery network [22], since like any other crowdsourced service the amount of people joining the system is one of the key parameters for such service to succeed.

We then use New York taxi data as opposed to using pedestrian data, since no known pedestrian-based dataset achieves the density and the number of users available in the New York City taxi dataset [22]. We highlight the fact that using taxis as carries does not affect our final results, since we focus mainly on relative time savings rather than absolute delivery completion times. We also note that studying our system on a dense and well established network such as those related to NYC taxis will provide optimistic results, which will be then considered as a best-case scenario for the ad hoc delivery system.

The second study we took into account balances instead the results of the first one, since we run the same experiments although on a more limited dataset, which comprises pedestrian and bicyclist data collected through OpenStreetMap in the area of the University of California, Irvine (UCI) campus, on a single day. We also highlight the fact that although our aim with these study is to show that such networks can be deployed in very different scenarios, clearly the NYC network would have better results, as deploying such networks takes time especially in recruiting users.

### 3.1. Approach

**Figure 1:**
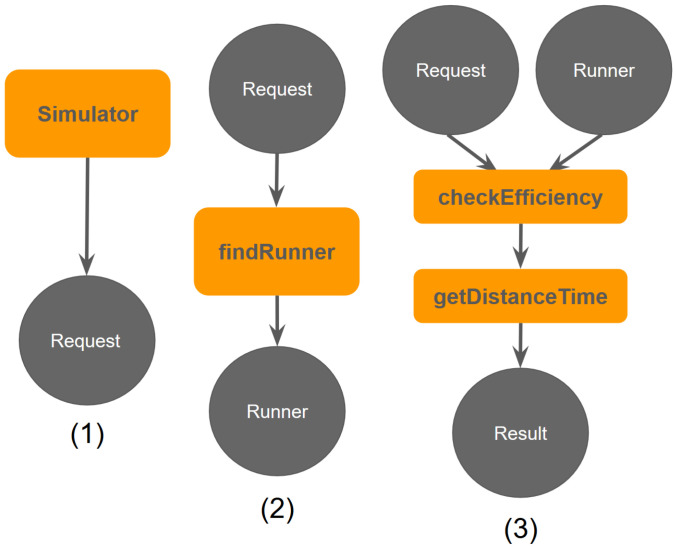
(1) The simulator generates a random request. (2) Processing by the delivery system to produce a carrier. (3) When the delivery is completed, efficiency metrics are computed [21] ©2020 IEEE.

In order to perform our study, we have developed a simulator built for the purpose of this study, which takes into account all the needed modules to run the experiments [21]. We present the architecture of our simulator in Figure 1.

As a first step, our simulator takes as an input a single delivery request made by a user. The request contains the current time, and the starting and ending location. In the following step the simulator needs to find the most suitable carrier for it in real time, accounting for the request characteristics and the carrier availability. Clearly the suitability of a carrier may be computed in different ways, such as minimizing the delivery distance, the delivery time, or achieving the minimum deviation from the original intended route. In our work, we focus on minimizing both the delivery time and the deviation time for the carrier. More formally, let *i* be the *i*-th available carrier and *j* be the *j*-th delivery request. We then compute the following linear combination for each available carrier F(i,j):

(1) $$F\left(i,j\right)=\gamma \cdot D\left(i_{s},j_{s}\right)+\left(1-\gamma \right)\cdot D\left(j_{e},i_{e}\right),$$

where $D(i_{s},j_{s})$ is the time that the carrier needs to go from its original location $i_{s}$ to the delivery pickup $j_{s}$, $D(j_{e},i_{e})$ is the time needed for the carrier to go from the delivery drop off location $j_{e}$ to its original destination $i_{e}$, and γ is a parameter which can be used to prioritize users and carriers for different maximum delivery and deviation times. Obviously when we are able to minimize $D(i_{s},j_{s})$, then we also minimize the delivery time, since the pickup occurs close to the original carrier route, hence reducing the total deviation time. Finally, $D(j_{e},i_{e})$ accounts for the remaining deviation time, since the carrier needs to travel from the delivery drop off location to its original destination. We eventually pick the *i*-th carrier which minimizes F(i,j) to deliver delivery request *j*. We also note that the carriers can only perform one delivery at a time: this means that when we select a carrier, such carrier is not able to serve any further delivery request.

To compute paths and metrics we rely on the Google Directions API, which accounts for different transportation modes such as walking, bicycling and driving. To ensure that the delivery requests are feasible, i.e., they do not fall in inaccessible areas such as bodies of water, we validate the request coordinates using MapQuest’s reverse Geocoding API (https://developer.mapquest.com/documentation/geocoding-api/, accessed on 1 December 2021).

Finally, we introduce the *willingness to wait* and *willingness to divert*. The success of the delivery system hinges on the user’s willingness to wait for a delivery and the carrier’s willingness to divert from a route to fulfill the delivery. We observe that in an efficient system, the majority of deliveries will be completed faster than users can complete them on their own.

### 3.2. Data

In this section, we detail the structure and the size of the datasets we have used in our study. As we already mentioned, we focused our efforts on two different scenarios, the first one in New York City, while the second one is constrained in the University of California Irvine (UCI) Campus.

#### 3.2.1. Nyc Taxi Traces

The NYC taxi dataset provides the GPS coordinates for taxis driving in the city at any time. Data are available since many years; however, to keep our study more tractable, we centered it on December 2013 [22]. We performed an initial cleaning of the data, by removing all those entities which were malformed or which generated or ended outside New York city. After the data cleaning, we are left with 12,888,814 total taxi journeys, averaging roughly 400,000 unique taxi journeys per day.

It is also important to note that the area of NYC is large, so we split the whole dataset into smaller sections which represent neighborhoods and smaller areas within Manhattan. The different areas we have used for our study can be found in Table 1. To make the analysis between different areas comparable, we also leverage the Layout Weighted Density metric, which was originally proposed in [22], as it better describes how the urban dynamics affects the routes. The LWD metric provides a standardized, quantitative method of describing a region’s road interconnectivity. The topologies of urban areas and other pedestrian-friendly areas like college campuses generally feature a higher number of road intersections and segments, which means greater interconnectivity, and a higher LWD score.

#### 3.2.2. Uci Campus Data

Compared to NYC, there was no published dataset of walker, runner, and bicyclist GPS route points for the UCI campus, hence we obtained a total of 91 GPS routes from OpenStreetMap contributors that corresponded to popular student walking and biking routes. The routes were completely anonymous and had no timestamps, so to make them ready to use for our study we randomly generated timestamps for them, distributing the routes according to the UCI class schedule. More precisely, we added timestamps to routes entering UCI so that they end at approximately 10 min before a typical class start time, and routes moving away from UCI are started approximately would start approximately 10 min after the end of a a typical class. To augment the data in our dataset, we also collected 13 further routes from Strava between October and December 2018, which brought the UCI dataset to a total of 104 different routes.

To make the two datasets more comparable, we have also subsampled the NYC dataset accounting for the same population density of the UCI dataset. Manhattan is 59 square km large, and in such areas there are around 6780 unique routes per square km per day. For the UCI dataset instead we have a 2.95 square km large area. The UCI populatino is made by over 36,000 students, which ends up in having over 12,203 students per square km. Finally, a trip onto campus and a trip off campus are accounted as two separate routes, so we can assume that around 6780 unique journeys can be found in UCI per square km per school day on UCI campus.Having computed these numbers, we are then able to fit the NYC dataset to the UCI dataset: we do so by sampling 2080 unique taxi routes, since such amount of routes over the 59 square km area of Manhattan end up in 35 unique routes per square km density, which is the exact same density we can found in the UCI dataset.

## 4. Numerical Results

In this section, we analyze the feasibility of our proposed network leveraging the dataset we have just presented. As a first step, we now define the different metrics we will use to evaluate the system in its different conditions for both NYC and UCI.

### 4.1. Metrics

- **Capacity Ratio**: this metric is computed by dividing the number of delivery requests in the system by the number of available carriers; the metric provides a universal expression of how much demand the delivery system is currently handling.
- **Success Ratio**: this metric represents the ratio of requests which are successfully served by our proposed system. In other words, whenever a request is issued, in case a carrier is available then the request is successful, otherwise it is not.
- **Average Time Saved By Users**: this metric studies the time saved when using our system compared to traditional user pick up, in which the users goes from the request coordinates to the delivery source and back.
- **Average Time Added to Carriers**: this metric analyzes the time added to carriers as they have to deviate from their original route, before eventually reaching their original intended destination.
- **Average Effective Time Saved By Users**: this metrics focuses on the difference between the user which goes to collect its parcel compared to the case in which the package is collected from the carrier.
- **Average Total Time Saved**: finally this metric computes the difference between the time which the carrier adds to its journey and the time that users need to collect the package on their own.
- **Effective Time Saved Ratio**: this metric is defined as:
  (2) $$\mathrm{ETSR}=\frac{\mathrm{UPT}+\mathrm{ETS}}{\mathrm{UPT}},$$
  where UPT is the User Pickup Time and ETS is the Effective Time Saved.
- **Total Time Saved Ratio**: this metric shows the ratio of additional time needed to perform the delivery and it is defined as:
  (3) $$\mathrm{TTSR}=\frac{\mathrm{UPT}+\mathrm{TTS}}{\mathrm{UPT}},$$
  where TTS is the Total Time Saved.

As a final note to the metrics we use, we also need to adjust each metric accounting for different carriers, so we also define the Adjusted ETSR and Adjusted TTSR.

(4) $$\mathrm{Adjusted}\;\mathrm{ETSR}={\left(\mathrm{ETSR}\right)}^{\alpha }\cdot {\left(\mathrm{Success}\;\%\right)}^{1-\alpha }$$

(5) $$\mathrm{Adjusted}\;\mathrm{TTSR}\;={\left(\mathrm{TTSR}\right)}^{\alpha }\cdot {\left(\mathrm{Success}\;\%\right)}^{1-\alpha }$$

If we then combine the adjusted TTSR with the adjusted ETSR, we eventually get the Delivery System Efficiency Rating (DSER), which is a single metrics which enables the comparison of different networks at once, already accounting for the heterogenity of the network.

(6) DSER=β·ETSR+(1−β)·TTSR

We note that α and β can be parameterized for additional flexibility in prioritizing the Success Ratio, TTSR, or ETSR. In our paper, we set α=0.9 and β=0.5, which emphasizes time savings over total completions without unfairly extracting time savings from either the carriers or the users. A deeper study on the effects of different values of α and β is left as future work.

### 4.2. New York Taxi Study Results

We now present the results for the first set of simulations. Clearly, for NYC the high density and plenty of availability of routes provide an excellent ground for an ad hoc delivery network, hence the system runs in an efficient way.

The efficiency results found in Table 2 were also in line with expectations. Even when running 10,000 requests (2.5% of the taxi system’s capacity), the delivery system saved users an average of 8.96 min and a total driving time of 10.78 min.

**Figure 2:**
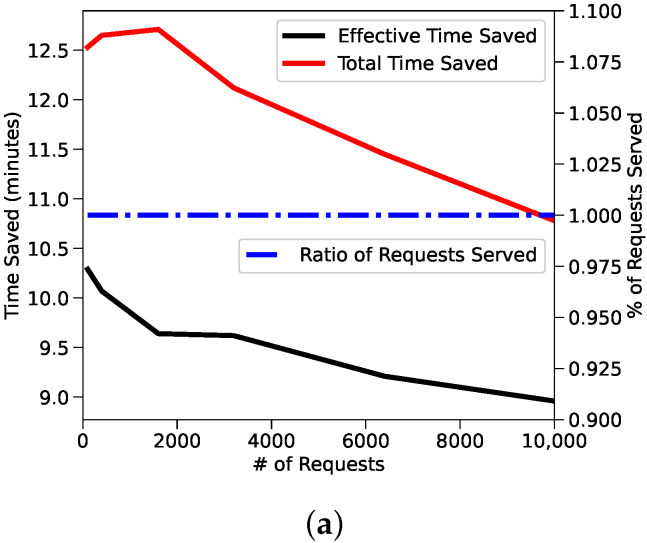

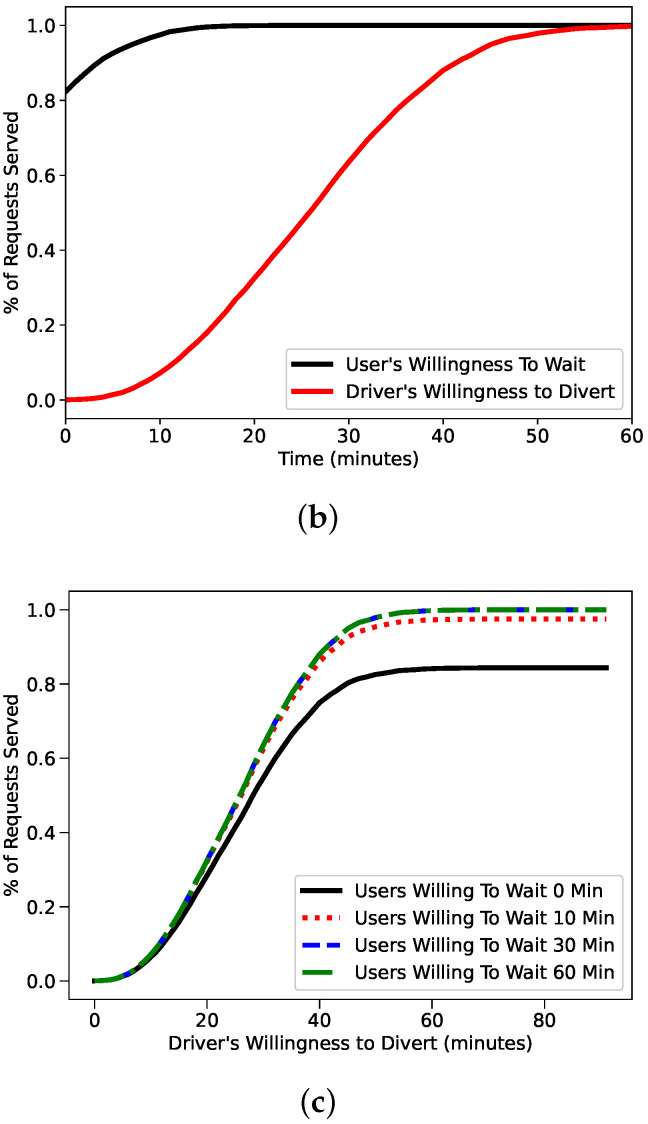
(**a**) presents the loss of as number of requests increase in Manhattan, New York. (**b**,**c**) highlight impact users and drivers willingness to wait drivers willingness to divert [21] ©2020 IEEE.

We can see in Figure 2a that the different metrics we study decrease when we increase the number of requests, however the percentage of requests served remains at 100%. This is expected, since the performance of our system is inversely proportional with respect to the number of requests issued. Clearly when the system starts to drop requests, the efficiency metrics would increase, but while the system continues to serve requests the performance degradation is achieved at the expense of efficiency.

Figure 2b shows instead the user willingness to wait; in case such willingness reaches 20 min, the performance of the delivery depends on the driver’s willingness to divert.

### 4.3. University of California, Irvine Study Results

The initial results reported in Table 3 were not promising. Even at less than 1% capacity (1 request), the delivery system required users to wait an average of 3.47 min. The total efficiency results were even worse as an average of 7.53 min were lost by users and runners per route. The poor results revealed an interesting nuance to crowdsourced delivery networks. The networks have difficulty supporting very short deliveries, i.e., when generating random delivery requests in a 2.95 square km campus, the delivery requests that cover less than 1.5 km in length are almost always better off being completed by the users themselves. Fortunately, we can assume that most delivery requests will be greater than 1.5 km in length since users will be less incentivized to utilize a delivery network when the pickup location is just a few minutes away. This issue did not affect the previous New York taxi study because the random delivery requests were generated in such a large area that most requests covered large distances. The sparse data in the study also saw runners being pulled from far distances to service requests when runner supply dwindled. The popular ride-sharing app Uber scans the user’s immediate vicinity for drivers and raises an exception if no drivers are found a certain distance away. This issue did not affect the New York taxi study because the supply of taxis is so dense that a taxi could always be identified a reasonable distance away from the user. We decided to mimic Uber’s logic and implement a runner bounding box. We wanted our bounding box to maintain a high success percentage and filter out only the most extreme outliers, so we set it at a generous 4 km (relative to the 2.95 square km campus).

Since the UCI area is much smaller compared to NYC, we set the minimum request distance to 1.5 km. This is because shorter requests are actually better completed by the users themselves, rather than asking a carrier to do it on their own. This allows us to center our analysis on the carriers, rather than incorporating results for routes which in a real scenario would be probably be completed by the users themselves.

After setting the minimum request distance to 1.5 km and the runner bounding box to 4 km, the results improved dramatically. As shown in Table 4, for a small decrease in success percentage, the efficiency of the system increased substantially. In the 1% capacity case, users went from losing 3.47 min on average to gaining 4.23 min. In the 50% capacity case (52 requests), we saw an even more substantial improvement. The system went from losing users an average of 6.95 min to gaining users 5.11 min. This came at the relatively small expense of dropping an additional 8% of requests. Compared to the 2.5% capacity case in the New York taxi study, which produced an ETSR of 1.242, the 2.9% capacity case of the UCI study produced an ETSR of 1.096.

**Figure 3:**
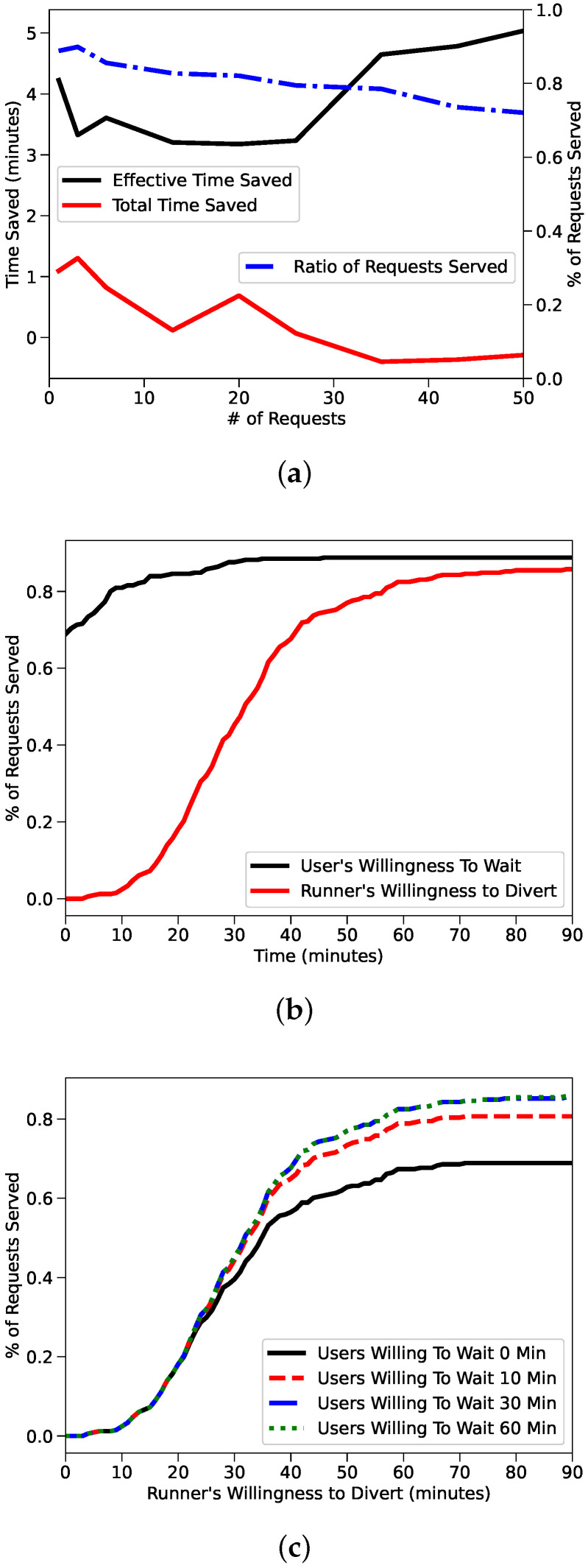
(**a**) presents the loss of as number of requests increase in the UCI campus. (**b**,**c**) highlight impact users and drivers willingness to wait drivers willingness to divert [21] ©2020 IEEE.

In the UCI study, we can see more dropped requests as the number of requests increase compared to the NYC study. This is evident in Figure 3a, where it is also clear how dropping inefficient requests may eventually lead to efficiency improvements. We also note that the NYC New York taxi study had higher TTS than ETS while in the UCI study we observe higher effective time savings. By favoring users over runners as we pointed out in Section 3 we expected to boost our effective time savings at the cost of total time savings, which makes the New York taxi results interesting. Finally we can state that the taxi supply in NYC is so extended that most of the deliveries can be completed with little deviation from the original route. Clearly this cannot be said for the UCI campus, as carriers need to divert more from their original route to fulfil the requests made by users. It is obvious that it is always possible to tailor the system to the specific needs, for instance to favor runners in case their willingness to participate in the network is proven to be a challenge, which is that Figure 3b,c show. Ideally, we would just increase the supply of runners and begin to see similar effective time savings and total time savings as in the New York study. We refer to Section 4.4 for a discussion as to whether or not a proportional increase in runners on the UCI campus could realistically lead to New York’s efficiency metrics.

We also review a more detailed breakdown of willingness on the 1 request case. Studying the 1 request case is equivalent to studying the case where there is an infinite supply of runners on fixed routes, that is, no runners are ever blocked servicing other requests. Like the New York study, we show that as long as users are willing to wait 20 min, the maximum % of requests served can be achieved. In this case, runners must be willing to divert more than than 80 min to reach the almost 90% of requests, but as long as runners are willing to divert greater than 40 min, almost 75% of requests can be fulfilled.

### 4.4. Uci-New York Bridge Study Results

In this section, we provide a comparison between the two different datasets that we used. For sake of fairness, we set also in NYC the same constraints of a 1.5 km minimum request distance and 4 km driver bounding box for the smaller areas considered. For the whole Manhattan area we also set the minimum request distance to 1.5 km, but we increase the driver bounding box to a more realistic 9 km. The purpose of this study is to be able to compare two extremely different scenarios, which show the flexibility of the system we propose. This would also serve as base for a preliminary performance assessment of our system in novel scenario.

**Figure 4:**
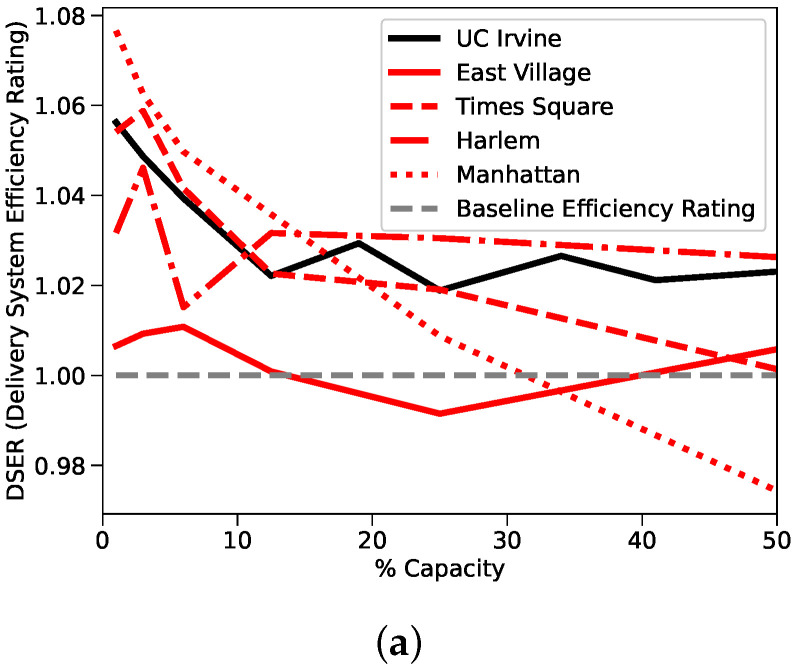

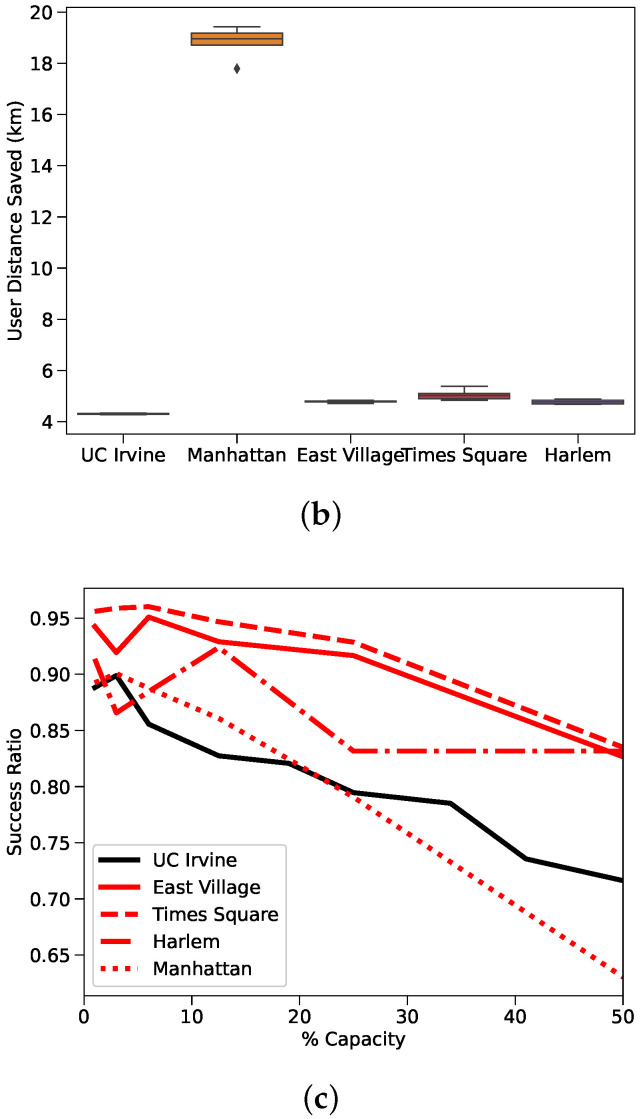
(**a**) presents a comparison between the UCI campus results and the four different areas extracted from the NYC dataset. (**b**) presents instead the average distance saved by all the users, while (**c**) analyzes the success ratio of the system [21] ©2020 IEEE.

In Figure 4a, we show the results of our comparison referred to the DSER metric which we have defined in Section 3, to highlight the resiliency of the system to high demands. In Figure 4b, we plot the average distance saved by the users for the 5 scenarios which we have taken into account. The fact that all the boxplots are quite thin refers to the fact that results are obtained with any system demand, since savings can be obtained even with larger demands. Alternatively, Figure 4c tracks the success ratio of the systems. Here, we can see that a larger capacity utilization eventually decreases the success ratio, since there are less carriers available to cope with the increasing delivery demands.

We also provide detailed results in Table 5, Table 6, Table 7 and Table 8 from the East Village, Time Square, Harlem, and subsampled Manhattan feasibility studies, respectively.

### 4.5. Results Analysis

#### 4.5.1. Similarity

The first observation is how similar the results between delivery systems are. All the measured scores fall between tight ranges; adjusted ETSR scores fall in a range between 1.02 and 1.15, adjusted TTSR scores fall between 0.88 and 1.04, and DSER scores fall between 0.97 and 1.07. Based on all the available performance metrics, the non-motorized UCI system behaves indistinguishably from the motorized NYC systems.

More advanced analysis reinforces the similarities. The relationship between the standard deviation in the route start/end times and success percentage persists across all the systems. The standard deviation in the route start/end times describes the coverage of carrier routes through the course of the day. The metric is calculated by first binning the number of route points into the 48 30-minute buckets spanning the day then computing the standard deviation on the number of route points in each bucket. Theoretically, a lower standard deviation would mean that the routes are well-spread out over the course of the day so that the success percentage should fall more gradually. In the higher standard deviation case, the success percentage falls with increased volatility as time buckets with low supply are exhausted. We see this phenomenon in the results as Manhattan and UCI, the owners of the highest standard deviations, suffer the most precipitous drops in success percentage.

#### 4.5.2. Efficiency

The second observation is that efficiency gains, while consistently positive, were ultimately slim. The average DSER of the five systems operating at a reasonable 20% capacity was approximately 1.018, which represents a 1.8% efficiency gain over a non-crowdsourced solution. The lack of significant efficiency gain indicates the challenges that face existing motorized, crowdsourced delivery solutions, especially in the urban, high-LWD regions we studied. Nevertheless, we highlight the fact that thanks to our solution it will be reduced the consumption and pollution due to the delivery itself. Moreover, it also allows users to receive parcels directly at their place, which could be key for a series of people with impaired mobility or for others with restricted movements or busy schedules. It is feasible that the high vehicular traffic commonly associated with dense, urban regions increases delivery times and decays efficiency. The fact that East Village, the studied region with the highest road interconnectivity and LWD score, had the worst DSER score supports this claim.

#### 4.5.3. Motorized vs. Non-Motorized

The non-motorized UCI delivery system, not only matched the performance of the motorized delivery systems, it outperformed. Given that the East Village system performed poorly with the highest LWD score, it makes some sense that the UCI system would perform well with the lowest LWD score. However, the UCI system relied completely on non-motorized traffic, which means the vehicular traffic discussed earlier would have had no impact on delivery times. Instead, urban areas with high LWD scores have typically been characterized as being more pedestrian friendly. Frequent intersections and short road segments typically improves pedestrian accessibility and pedestrian safety as it forces vehicular traffic to slow. It is reasonable to assume that for a zero-emission crowdsourced delivery system powered by non-motorized traffic, a higher LWD score would increase the efficiency numbers. Given this assumption, there must exist a LWD score, where a motorized delivery solution’s performance is identical to that of a non-motorized delivery solution. Results from the bridge study appear to place this score between 0.028 and 0.032.

In conclusion, the bridge study effectively closed the gap between the New York and UCI studies and demonstrated that a local, motorized New York system could serve as a realistic proxy for a non-motorized UCI campus delivery system as long as the local system encompassed a region with a LWD score around 0.03. Higher LWD scores appear to favor non-motorized delivery systems and lower LWD scores favor motorized systems.

## 5. Route Prediction Study

In this section, we propose and evaluate a methodology to predict future routes of carriers. This would allow to know in advance possibilities for deliveries, hence making it possible to forecast service performances.

To make deliveries more seamless for carriers, we propose three system upgrades:

1. *Multiple Deliveries*: Instead of blocking a carrier’s route once a request is accepted, update the carrier’s route dynamically to include the accepted delivery request. This allows multiple deliveries to be made on the same route.
2. *Relays*: Compute multi-carrier routes that include efficient “handoff” locations, where carriers can pass off a package to another carrier.
3. *Route Prediction*: Adjust carrier routes to maximize the possibility of receiving delivery requests by leveraging historical delivery data.

Of these system improvements, route prediction offered the clearest cost-benefit picture. For instance, enabling *multiple deliveries* increases the delivery time of an original request every time a new request is accepted. The *relay* system would in theory only make the system more efficient, but every time a “handoff” is scheduled, an unknown is introduced into the system; there are no guarantees that two unfamiliar carriers will identify themselves in an efficient manner. We proceeded to build a *route prediction* model, which we will cover in the next section.

We once again used the New York taxi dataset from [22] for our route prediction task. We chose the dataset for consistency reasons and because of the dataset’s relatively short date coverage. The dataset only covers New York taxi trips that occurred between 1 December 2013 and 31 December 2013. This sparsity would provide an interesting analogue for the task of providing a route prediction service at the early stages of the delivery network’s development. We also considered the possible memory and computational savings of a machine learning model compared to a model based on historical averages.

### 5.1. Approach

The general approach of the route prediction model was based on [23], from which we adapted the following techniques to our study:

1. Time was binned in 30 min increments.
2. Dates were binned by day of week.
3. A representational value between 0 and 1 was computed for each time and date bin.
4. Each time and date bin value was further processed with *Sine* and *Cosine* functions.
5. We differentiate between weekdays and weekends.
6. A geohash was computed in order to uniformly bin longitude/latitude coordinates.

Further details on how we process the data are as follows:

1. A *holiday* label was added in addition to the *business day* in order to account for holidays. We considered three cases: the 0 holiday case, 1 holiday case, and 4 holidays case. The 1 holiday case would follow the federal calendar and recognize Christmas day (12/25) as the only holiday in December. The 4 holiday case would follow some state calendars and recognize Christmas Eve (12/24), Christmas Day (12/25), Day After Christmas (12/26), and New Years Eve (12/31) as official holidays.
2. The original work aggregated the number of pickups as a count. This assumes that each bin is represented equally in the data. Since we only have one month worth of data and we are accounting for holidays, we instead aggregated the number of pickups using a mean.
3. We considered replacing the use of geohashing with MGRS. MGRS offers more precision guarantees when defining its area representations. For example, a precision of 3 produces a 100 square meter area while a precision of 4 produces a 10 square meter area. This precision extends anywhere in the world. Geohashing is not intended to offer the same precision guarantees as MGRS. When its precision is set to 7, it represents around a 76 square meter area on the equator; however, in NYC, that region is around 118 square meters.

Our approach also differed from the previous work in that the previous work evaluated the route prediction models using a generic 80/20 training/testing split [21], while we will also be performing this test, we are extending the evaluation to include a more realistic route prediction test, i.e., we will attempt to predict the following day’s pickups based on historical pickup data. Specifically, we will generate predictions for each day based on the accumulated historical statistical data of previous days. To convey how machine learning may help or hurt the task, we will use a baseline model that makes predictions based on historical averages and compare the results to those of our trained models. Three versions of the baseline model were also created, as weekdays may vastly differ from holidays or weekends. Each version we propose accounts for 0, 1, or 4 holidays, respectively. To prevent overfitting and return zero results, we only grouped by the *business day*; in other words, data from holidays and weekends were pooled into the same bucket.

To evaluate the models, we will use the two empirical metrics, R^2^ score and RMSE score. In the case where baseline predictions differ significantly from model predictions, we will also leverage heatmaps to perform qualitative analysis.

For our initial analysis, we used a Random Forest model, which we trained accounting for a different number of holidays.

### 5.2. Randomized 80/20 Split Evaluation

The purpose of the initial randomized 80/20 split evaluation was to evaluate MGRS against geohashing. The R^2^ score and RMSE score of the MGRS-based models consistently trailed those of the geohash-based models by small margins. Our results indicate that geohashing and MGRS decode and encode with the same amount of noise. In addition, geohashing produces a more optimal area representation size. (118 square meter vs. 100 square meter). As areas reduce down to 0 square meter blocks, they no longer encompass definable regions of the city. Furthermore, as areas increase, they may become too large and definable regions of the city bleed into each other. Outside of this intuition, limited work has been done on optimal geography representations for ML. It is import to also consider MGRS’s precision guarantees; it represents the same area size anywhere in the world while geohashing is affected by the curvature of the Earth. We considered the precision guarantees, but since our study was local to NYC, they were not prioritized. We opted to prioritize prediction accuracy, and removed the MGRS-based models from later tasks. We summarize our results in Table 9.

### 5.3. Next Day Evaluation

**Figure 5:**
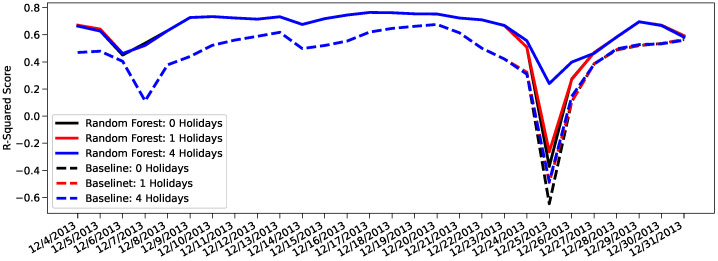
Next day prediction results between 4 December 2013 and 31 December 2013 using Random Forest and Baseline models trained with 0, 1, and 4 recognized holidays.

WThe next day prediction results for 4 December 2013 to 31 December 2013 using the 3 baseline models and 3 random forest regression models were graphed in Figure 5.

The three dates that show the most unique behavior are 7 December 2013, 25 December 2013, and 31 December 2013. On 7 December 2013, we see the model prediction improve from the previous day’s prediction while the baseline prediction worsens. This happens as the model prediction has now learnt the mobility behavior of NYC thanks of the training on the previous days, while the baseline model only accounts for the average of vehicles. On 25 December 2013, all the models see significant drops in their R^2^ score; the only model that persists some predictive ability is the 4-holiday Random Forest model, which better describes the specific nature of Christmas day. Finally 31 December 2013 represents new year’s eve, which although a festive day, it also experiences a raised demand in taxi trips.

**Figure 6:**
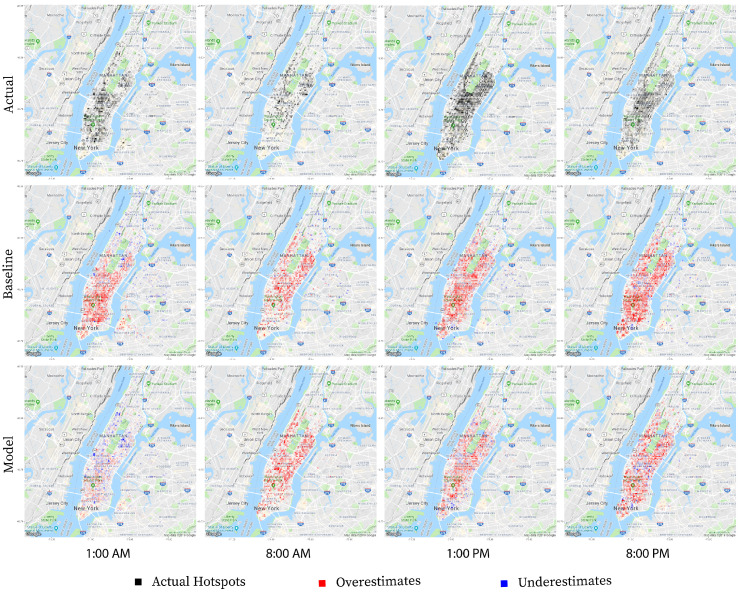
A comparison between the baseline prediction, 4-holiday Random Forest model prediction, and actual result for next day prediction of Christmas day 25 December 2013.

Christmas day or 12/25 provides an interesting study because every model but one predicts worse than random. To understand why this is happening, we once again review the basis of the predictions. To prevent overfitting, the baseline model groups holidays and weekends together as *non-business days*. The 4 holiday baseline model specifically averages the results from Christmas Eve or 12/24 and results from previous weekends (12/1, 12/7–8, 12/14–15, 12/21–22) to attain the predicted results for 12/25. The 4 holiday random forest regression model has access to the business day information, but reinforces the relationship between Christmas day and Christmas Eve with an additional *holiday* label. Figure 6 illustrates how the model takes the additional information into account. At 1:00 a.m., the baseline model overestimates the number of taxi pickups because it assumes behavior similar to the typical Saturday 1:00 a.m. and Sunday 1:00 a.m. when in reality, the taxi traffic on the night before Christmas day shares more in common with a typical Monday at 1:00 a.m. The 4-holiday random forest regression model leverages its knowledge of Christmas Eve’s relationship with Christmas Day to learn that taxi traffic typically shrinks on the night before a holiday. As the day progresses, both the baseline and the model continue to overestimate with the baseline consistently overestimating more than the model.

### 5.4. Route Prediction Implementation and Evaluation

Having successfully demonstrated our ability to produce next day taxi pickup predictions using a random forest regression model, we proceed to implement a basic version of route prediction in our simulator to test its impact on our efficiency metrics. In the route prediction simulation, a driver wakes up at a random time and a random location with a random destination. Prior to departing for the random destination, the driver checks the route prediction model to locate an area with high taxi pickup requests. The driver will deviate at most 4 km from the original route to reach the area of increased pickup activity. If the route prediction model returns a valid waypoint, the driver departs immediately for the waypoint without waiting 15 min as described in Section 3.2 and waits 15 min at the waypoint instead. If the model does not return a waypoint, the driver waits 15 min at the starting point before departing for the destination as per usual. Furthermore, unchanged, the driver continues to wait 15 min at the destination before ending the route.

This approach is intended to proxy the carrier experience in a zero-emission crowdsourced delivery system. The carriers power on their apps when they are ready to go out for a walk, run, or bicycle ride. The carriers are willing to wait up to 15 min to either service a valid delivery or be recommended a route with a high probability of a delivery request. The carriers are also willing to wait another 15 min at their final destination.

One final distinction is that the new approach randomizes the driver routes and uses the routes in the data as pickup routes. This is opposed to the previous approach of using the routes in the data as driver routes and randomizing the pickup requests. Randomized driver routes tax the delivery system less than randomized pickup requests. The observation is actually consistent with the analysis made in Section 4.5. Having low standard deviation in the driver routes produces more gradual degradation in the success percentage, which contributes to more robust efficiency. By randomizing the driver routes, we ensure a low standard deviation. The observation is also partially explained by the fact that randomized driver routes allow for less entropy than randomized pickup requests. Randomized driver routes are generated by randomly choosing two coordinates and populating the route with the Open Source Routing Machine (OSRM), which on average, generates 60 coordinates per route. Even if the start and end coordinates represent outliers, the probability that a significant number of the 60 coordinates falls in a popular pickup area remains high. On the contrary, it is far easier to generate random pickup request locations that fall outside of popular driver routes.

### 5.5. New York Taxi Study Results

**Figure 7:**
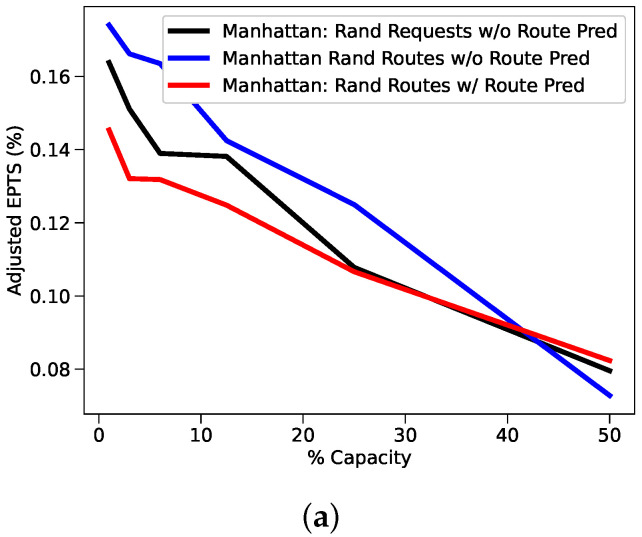

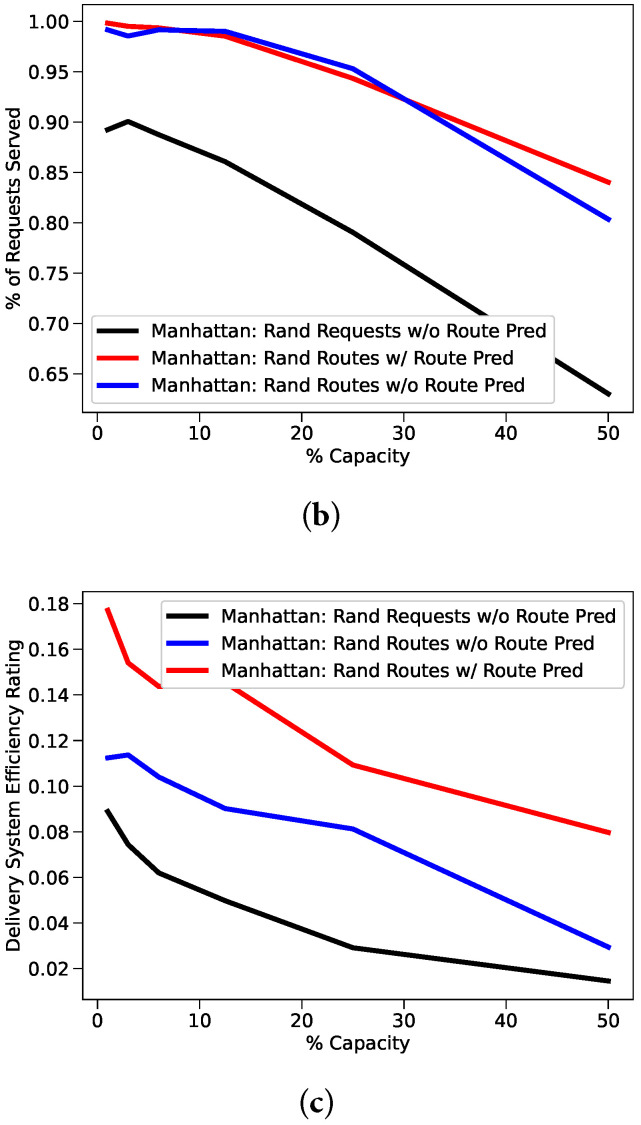
A comparison between three subsampled Manhattan studies. (**a**) The first study disabled route prediction, randomized pickup requests, and generated routes from taxi data. (**b**) The second study disabled route prediction, generated pickup requests from taxi data, and randomized routes. (**c**) The third study enabled route prediction, generated requests from taxi data, and randomized routes.

Figure 7 illustrates both the benefits of using route prediction, and the impact of depressed standard deviation and entropy with randomized driver routes. Results with randomized driver routes and route prediction disabled are significantly better than those from the original feasibility study, but also significantly trail the results with route prediction enabled. In the 50% capacity case (1040 requests), adding route prediction helped maintain positive values for effective time saved, total time saved, and total distance saved. Seeing a total time saving of almost 3 min was especially impressive given that the original run lost 3 min in the same 50% capacity case. Furthermore, noteworthy is the increase to total time savings. When concluding Section 4.3, we compared the results from the initial New York feasibility study and the UCI feasibility study and noticed consistently higher total time savings than effective time savings in the New York study. We posited that the large number of taxi routes being driven at any given moment allowed the delivery system to choose on-the-way deliveries for drivers so that additional routing was minimized. Once we began subsampling, the number of taxis and constraining our systems in our bridge studies, we no longer could reproduce this result. However, after implementing a basic version of route prediction, we once again observe higher total time savings than effective time savings despite the identical constraint of 2080 taxi routes. Increasing the total time savings means we are minimizing the carrier divert time, and increasing the overall efficiency of the delivery system, which we can clearly see as the route prediction increases efficiency by almost 12% over the baseline model.

To fully understand the value of route prediction, we need to reproduce the remaining Section 4 analysis with route prediction enabled. Reductions to carrier willingness and user willingness will need to be studied, and the route prediction model will need to be tested in UCI as well as every subsampled NYC region.

## 6. Real World Extension Proposal

In this section, we describe a proposal for a real-world extension of our framework, by designing a mobile application which offers the same services as we described.

**Figure 8:**
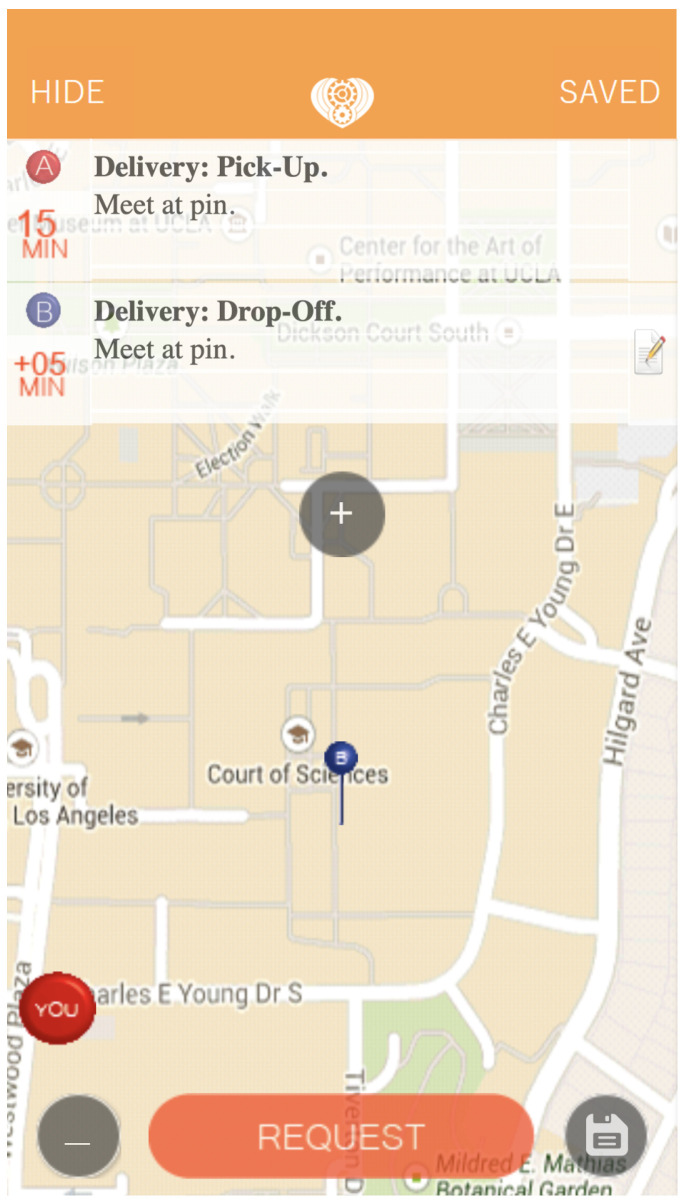
A screenshot of a prototype mobile app that enables the zero-emission delivery transactions.

A first aspect to take into account is about the LWD score: as we have shown, the LWD score has a key role to determine the feasibility of such networks, therefore the system must be implemented in a sufficiently dense area, i.e., locations with a LWD score greater than 0.028. We have also reviewed potential improvements to the system, and implemented a basic version of route prediction. The logical next step is to propose a real-world study. The framework proposed in Figure 1 is already fully-equipped to handle real-time requests, also considering possible scalability problems. The *findRunner* module identifies the best carrier based on pickup coordinates, dropoff coordinates, and a timestamp. Staging this framework for the real world would require developing a mobile app that can be used by both users and carriers. We have prototyped a preliminary version of the app in Figure 8. When carriers log onto the app, their carrier id will be logged in a database so that they can be queried when a delivery request is scheduled. Deliveries that then come through the app will be processed by the *findRunner* module, which queries the database, and returns the active carrier best suited for the job. Once the user and the carrier accept the delivery, the delivery is tracked in real-time on a map. Time and distance metrics are recorded for consumption by the *checkEfficiency* module to better understand the performance of the app and for possible future improvements. The same analysis above could then be run on the real-world data and compared with the simulated results.

One careful consideration remains before a real-world extension is finalized, since real-world carriers will require rewards for their work, hence incentives: we address this challenge in the next section.

### Incentivisation

Incentivising carriers to divert from their original paths to complete deliveries is and will continue to be one of the greatest challenges facing our delivery system. All currently available crowdsourced systems implement a payment system for the exchange of monetary value between the *servicer* and the *servicee* [2,21].

Our goal is to present such a seamless experience that carriers performing deliveries add negligible time to their routes. We strive to present carriers with an experience less akin to the ridesharing app Uber and more akin to the navigation/traffic avoidance app Waze, where the role of *servicee* and *servicer* is integrated. Anyone who opens Waze is seamlessly *producing* real-time traffic data in the background while *consuming* navigation directions in the foreground. The onus is on us to produce a system so seamless for carriers that further incentives are unnecessary, since users are willing to contribute to the app because it provides a useful service also for them when they will need deliveries on their own.

Recall that our system did not reach its peak performance until carriers’ willingness to divert reached 60 min. The improvements we propose in Section 5 including route prediction, will bring this number down, but not to zero. Our far more realistic goal is to reach peak performance when carriers’ willingness to divert reaches 30 min, which would however cover a large portion of requests. The next part of our solution is re-framing the application itself. Instead of treating carriers like the *servicer*, we intend to treat carriers like *fitness app users*, i.e., carriers will only log onto the app when they are about to go on a walk, run, or bicycle ride for the sole purpose of physical activity. The app will operate similarly to existing fitness tracking and fitness community apps. This way, diverting from an original path is acceptable as long as it is within a certain pre-specified window of time and the physical activity of choice can be performed. It is our job to ensure that each delivery remains within the pre-specified window of time and maximizes the physical activity of choice.

By translating the physical activity from something the carrier *must* do to something the carrier *wants* to do, we hopefully lower the need to incentivize carriers. The only thing left to incentivize is the time the carriers spend away from their physical activity of choice while completing the delivery. Our initial attempt at a solution will not use traditional monetary exchange to incentivize this; instead, the solution will allow users to donate arbitrary amounts to charities pre-specified by the carriers. Users can then learn about local nonprofit causes and express their gratitude while incentivizing carriers for the overhead delivery time.

## 7. Conclusions

In this work, we presented a feasibility study on two real-world datasets for a crowdsourced delivery network, using existing opportunistic carrier paths. Our system shows good performance in different scenarios, both in terms of motorized and non-motorized carriers, and under different urban dynamics. As it has been shown, the LWD score, and in general the density of potential carriers plays a paramount role for the success of the proposed solution. The results indicate that such a network is indeed feasible, and can provide benefits both in terms of time saved by users and emissions reduced, as we leverage existing routes traveled by carriers instead of adding new ad hoc ones. We have then leveraged our promising results to motivate a real-world study while proposing a solution to the incentivization challenge, and demonstrating how technological improvements like route prediction further decrease the burden on carriers while producing faster deliveries for users.

### Future Work

Future work on this topic includes fully developing the route prediction study and investigating additional technological improvements like multiple delivery support and relay functionality, as well its comparison with other similar deployed services. We intend to demonstrate how further improvements increase the efficiency and capacity of the current delivery system before developing a mobile app prototype to enable real-world deliveries. We also aim to work on the metrics which are used to select carriers, as they have a direct impact on the performance of the system. This includes the study of novel metrics, as well as optimizing the current ones. Finally, we aim to run the real-world prototype delivery system in an area with high road interconnectivity and a high LWD score. We would then collect additional efficiency metrics and determine the real-world applicability of green crowdsourced social delivery networks. This will also open up the possibility to work on the foreseen incentive techniques and test their validity.

## Figures and Tables

**Table 1:** Various regions of Manhattan and corresponding datasets compared to the UCI campus and datasets.

Location	Avg Street Len (m)	*LWD* Score	Avg Route Time (min)	Route Start/End Times Std Dev
East Village, Manhattan, NY	40.6826	0.0530	11:20 ^1^	189.08
Manhattan, NY	60.2037	0.0331	10:53 ^1^	1486.03
Times Square, Manhattan, NY	63.0989	0.0319	13:07 ^1^	127.60
Harlem, Manhattan, NY	62.9538	0.0308	9:21 ^1^	130.94
UC Irvine, Irvine, CA	60.8222	0.0284	47:28 ^2^	1491.29

^1^ Motorized traffic, ^2^ Non-motorized traffic.

**Table 2:** Results from New York taxi feasibility study.

% Capacity	# Requests	% Success	Runner Time Added (min)	Runner Distance Added (km)	Effective Time Saved (min)	Total Time Saved (min)	Total Distance Saved (km)
**0.025%**	100	100%	24.23	9.35	10.29	12.53	7.75
**0.1%**	400	100%	24.51	9.78	10.07	12.65	8.14
**0.4%**	1600	100%	23.35	9.30	9.64	12.71	7.81
**0.8%**	3200	100%	24.82	10.12	9.62	12.12	7.60
**1.6%**	6400	100%	25.36	10.68	9.21	11.45	6.87
**2.5%**	10,000	100%	26.16	11.30	8.96	10.78	6.33

**Table 3:** Results from UC Irvine campus feasibility study.

% Capacity	# Requests	% Success	Runner Time Added (min)	Runner Distance Added (km)	Effective Time Saved (min)	Total Time Saved (min)	Total Distance Saved (km)
**0.96%**	1	95%	27.86	3.33	−3.47	−7.53	−0.89
**2.9%**	3	94%	28.93	3.44	−4.05	−8.21	−1.01
**5.8%**	6	93%	30.10	3.49	−3.99	−9.16	−1.05
**12.5%**	13	92%	30.62	3.53	−4.71	−9.93	−1.14
**25%**	26	91%	33.09	3.79	−5.79	−11.97	−1.35
**50%**	52	80%	37.54	4.13	−6.95	−15.72	−1.66

**Table 4:** Results from UCI campus feasibility study after restricting pickup requests to be greater than 1.5 km away and automatically dropping any request that can only be serviced by a runner who is more than 4 km away.

% Capacity	# Requests	% Success	Runner Time Added (min)	Runner Distance Added (km)	Effective Time Saved (min)	Total Time Saved (min)	Total Distance Saved (km)
**0.96%**	1	89%	33.51	4.29	4.23	1.10	0.05
**2.9%**	3	90%	33.32	4.44	3.32	1.30	−0.16
**5.8%**	6	86%	34.81	4.35	3.61	0.82	−0.04
**12.5%**	13	83%	35.62	4.36	3.20	0.12	−0.09
**25%**	26	79%	34.69	4.47	3.23	0.07	−0.17
**50%**	52	72%	37.72	4.56	5.11	−0.27	−0.23

**Table 5:** Results from East Village feasibility study.

% Capacity	# Requests	% Success	Runner Time Added (min)	Runner Distance Added (km)	Effective Time Saved (min)	Total Time Saved (min)	Total Distance Saved (km)
**0.96%**	1	94%	25.08	5.24	1.60	−0.93	−0.48
**2.9%**	3	92%	24.48	5.09	1.42	−0.46	−0.29
**5.8%**	6	95%	24.74	5.19	1.69	−0.84	−0.39
**12.5%**	13	93%	24.97	5.30	1.47	−1.02	−0.50
**25%**	26	92%	24.61	5.04	1.37	−1.36	−0.34
**50%**	52	83%	25.23	5.32	2.28	−0.91	−0.48

**Table 6:** Results from Time Square feasibility study.

% Capacity	# Requests	% Success	Runner Time Added (min)	Runner Distance Added (km)	Effective Time Saved (min)	Total Time Saved (min)	Total Distance Saved (km)
**0.96%**	1	96%	24.39	4.83	2.12	1.25	0.29
**2.9%**	3	96%	24.28	4.75	2.42	1.17	0.32
**5.8%**	6	96%	23.72	4.70	1.97	0.51	0.18
**12.5%**	13	95%	24.73	4.88	2.14	−0.62	−0.04
**25%**	26	93%	25.67	5.10	2.25	−0.76	−0.14
**50%**	52	83%	27.71	5.66	2.12	−0.94	−0.27

**Table 7:** Results from Harlem feasibility study.

% Capacity	# Requests	% Success	Runner Time Added (min)	Runner Distance Added (km)	Effective Time Saved (min)	Total Time Saved (min)	Total Distance Saved (km)
**0.96%**	1	91%	18.98	4.82	0.97	0.85	0.06
**2.9%**	3	87%	18.59	4.66	1.56	1.14	0.18
**5.8%**	6	88%	19.33	4.82	0.87	0.35	0.00
**12.5%**	13	92%	18.03	4.48	0.78	0.89	0.19
**25%**	26	83%	17.86	4.45	0.75	1.37	0.29
**50%**	52	83%	17.95	4.55	0.87	1.05	0.15

**Table 8:** Results from subsampled Manhattan feasibility study.

% Capacity	# Requests	% Success	Runner Time Added (min)	Runner Distance Added (km)	Effective Time Saved (min)	Total Time Saved (min)	Total Distance Saved (km)
**0.96%**	20	89%	40.06	18.01	7.47	0.64	1.41
**2.9%**	60	90%	40.82	18.02	6.83	−0.10	1.16
**5.8%**	120	89%	40.86	18.03	6.29	−0.68	0.70
**12.5%**	260	86%	41.97	18.60	6.45	−1.80	0.11
**25%**	520	79%	43.15	19.50	5.54	−2.54	−0.32
**50%**	1040	63%	42.90	18.77	5.02	−3.17	−0.98

**Table 9:** Results from randomized 80/20 split evaluation.

	Geohash				MGRS			
**Model Type**	**Train R^2^**	**Test R^2^**	**Train RMSE**	**Test RMSE**	**Train R^2^**	**Test R^2^**	**Train RMSE**	**Test RMSE**
**0 Recognized Holidays**								
**Random Forest**	0.9845	0.8896	0.0391	0.1044	0.9808	0.8615	0.0357	0.0959
**1 Recognized Holiday (12/25)**								
**Random Forest**	0.9819	0.8692	0.0423	0.1137	0.9779	0.8405	0.0385	0.1033
**4 Recognized Holidays (12/24, 12/25, 12/26, 12/31)**								
**Random Forest**	0.9785	0.8450	0.0468	0.1257	0.9741	0.8131	0.0425	0.1141
